# Stabilization of G-Quadruplex Structures of the SARS-CoV-2 Genome by TMPyP4, BRACO19, and PhenDC3

**DOI:** 10.3390/ijms25052482

**Published:** 2024-02-20

**Authors:** Miklós Cervenak, Orsolya Réka Molnár, Péter Horváth, László Smeller

**Affiliations:** 1 Department of Biophysics and Radiation Biology, Semmelweis University, 1094 Budapest, Hungary; cervenak.miklos@med.semmelweis-univ.hu (M.C.); molnarorsolyareka@gmail.com (O.R.M.); 2 Department of Pharmaceutical Chemistry, Semmelweis University, 1092 Budapest, Hungary; horvath.peter@semmelweis.hu

**Keywords:** G-quadruplex, SARS-CoV-2, RNA, oligonucleotide, FRET, FAM-TAMRA pair, spectroscopy, TMPyP4, BRACO19, PhenDC3

## Abstract

The G-quadruplex is one of the non-canonical structures formed by nucleic acids, which can be formed by guanine-rich sequences. They became the focus of much research when they were found in several oncogene promoter regions and also in the telomeres. Later on, they were discovered in viruses as well. Various ligands have been developed in order to stabilize DNA G-quadruplexes, which were believed to have an anti-cancer or antiviral effect. We investigated three of these ligands, and whether they can also affect the stability of the G-quadruplex-forming sequences of the RNA genome of SARS-CoV-2. All three investigated oligonucleotides showed the G-quadruplex form. We characterized their stability and measured their thermodynamic parameters using the Förster resonance energy transfer method. The addition of the ligands caused an increase in the unfolding temperature, but this effect was smaller compared to that found earlier in the case of G-quadruplexes of the hepatitis B virus, which has a DNA genome.

## 1. Introduction

**Figure 1:**
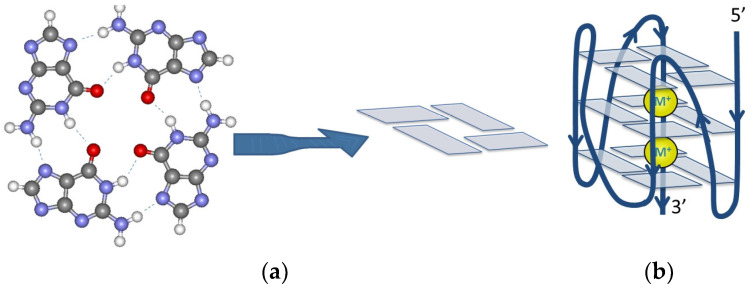
(**a**) Structure and schematic representation of a G-quartet composed of four guanine bases connected with Hoogsteen-type hydrogen bonds; (**b**) structure of a parallel GQ conformation. M^+^ represents the stabilizing metal ion, which is usually K^+^ or Na^+^.

The G-quadruplex (GQ) is one of the non-canonical structures formed by nucleic acids. GQs can be formed by guanine-rich sequences [1,2,3,4]. Four guanines are arranged in a planar structure stabilized by Hoogsteen-type hydrogen bonds, forming a G-quartet (Figure 1a). Two or three of such quartets compose a G-quadruplex (Figure 1b). Cations are also necessary to stabilize the GQ structure. These are either in the middle of the G-quartet or between them, depending on the cation size. Potassium and sodium are the most frequent stabilizing ions, although other ions like Rb^+^ and even bivalent ions have been also reported to stabilize GQs [5,6,7]. Potassium is the stronger stabilizing ion in case of most of the oligomers reported in the literature; however, exceptions to this rule have also been reported [5,8,9]. A GQ can be monomeric, dimeric, or tetrameric, depending on the number of the nucleic acid strands they are composed of. According to the orientation of the strands, GQs can adopt parallel, antiparallel, or hybrid structures [10,11,12]. The loops can be lateral, diagonal, or propeller-type. The actual conformation of the GQ depends on several factors, including the type of the cation [13,14], the concentration [15], loop size, and sequence [16,17]. Even if the conformation of the core of the folded GQ is the same, the stability can be influenced considerably by the ion type and loop length [5,17,18].

DNA GQs came into the spotlight of cancer research when they were found in the telomere region, and additionally in several oncogene promoter regions [19]. The presence of GQs in the telomere region of DNA can inhibit the telomerase enzyme, which elongates the telomere region in immortal (cancer) cells. In this context, the stabilization of GQs with special ligands appeared to be an important possibility in fighting against cancer [20,21,22,23]. Besides the telomere region, GQs have been identified in the promoter regions of known oncogenes or proto-oncogenes, which also appears to be a promising target of medical research [24,25]. GQ-binding proteins have also gained interest as potential drug targets [26].

Naturally, not only the human genome contains GQ-forming sequences. Several viral genomes were analyzed by Lavezzo et al. [27], who concluded that many viruses have more potential GQ-forming sequences than expected, simply from their nucleotide composition. Some of the predicted GQs were experimentally confirmed by other authors [28]. GQs in bacterial genomes present a route for potential antimicrobial therapy [29]. The importance of GQs as targets in antiviral therapy was pointed out by Ruggerio et al. [30,31]. In our previous papers, we analyzed three GQ-forming sequences of the hepatitis B virus [9,32]. We confirmed the formation of GQ structures in three possible GQ-forming sequences (PQSs). One of them is in the coding region of the polymerase protein; the other one can be found in the envelope coding gene of the S protein of the hepatitis B virus. The third one is situated in the C protein’s signaling region.

Several organic molecules have been developed in order to stabilize the GQ structure [33,34,35,36]. One of the most widely studied ligands is TMPyP4 (meso-5,10,15,20-Tetrakis-(N-methyl-4-pyridyl) porphine), which was proven to bind to telomere GQs and consequently to inhibit telomerase activity [37]. Recently, its effectiveness in suppressing viral infection and lung inflammation in animal models of SARS-CoV-2 infection was also demonstrated [38].

The two other ligands we studied were BRACO19 and PhenDC3. The trisubstituted acridine compound BRACO19 (N,N′-(9-(4-(Dimethylamino)phenylamino) acridine-3,6-diyl)bis(3-(pyrrolidin-1-yl)propanamide) hydrochloride) was also shown to inhibit telomerase activity in cells and tumor xenografts [20], and it has also been proven to exhibit antitumor activity [39]. It has also shown anti-HIV-1 activity, as demonstrated by Perrone et al. [40], who concluded that this effect is based on the strengthening of viral GQs by BRACO19.

The third ligand, PhenDC3 (3,3′-[1,10-Phenanthroline-2,9-diylbis(carbonylimino)] bis [1-methylquinolinium] 1,1,1-trifluoromethanesulfonate (1:2)), has been suggested for use in assays to indicate the formation of G-quadruplexes due to its high-affinity binding to GQs [41].

In our earlier studies on GQs in the genome of the hepatitis B virus, we determined the temperature stability of these GQs. TMPyP4, BRACO19, and PhenDC3 were found to stabilize these structures to different extents [9].

Numerous DNA-based GQs have been studied: their structure and their variants with different loop regions have been characterized [42,43], and their stability has been assessed in various environmental conditions, e.g., in molecular crowding [44]. Considerably less is known about the GQs formed by RNA strands, although some of them have been reported in the case of RNA as well [45,46].

Panera [47] analyzed the genome of the SARS-CoV-2 virus from point of view of possible GQ-forming sequences. Several short sequences were identified, which can presumably form GQ structures. Belmonte-Reche et al. included the extent of the conservation of the sequence in their analysis [48]. Their prediction overlaps partially with that of Panera, e.g., the oligomers with starting positions 13385 and 28903 are included in both lists. Despite the number of predictions made, very few of these predicted structures have been experimentally investigated. It has been pointed out that their interaction with small molecules and also with proteins is of great importance from a medical point of view [49]. Here, we investigate three oligomers from the first four described with the highest G score in ref [47]. We named them after the order of their appearance there. Also, two bases were added to the oligomers, in order to reduce the quenching effect of guanine on our fluorophores. The sequences are listed in Table 1. We selected originally the first four sequences, but our preliminary experiments showed that C19/2 does not form GQs, which is in agreement with the results published by Zhao [50]. This is why we left C19/2 out of our investigation.

The aim of this study is to characterize the stability of the oligonucleotides that were predicted to form quadruplex structures. We measured their thermodynamic parameters using the Förster resonance energy transfer method. The second question was whether RNA GQs can be stabilized by these ligands that were developed to stabilize the GQs in telomere and other DNA sequences.

## 2. Results

### 2.1. GQ-Forming Ability and Stability of the SARS-CoV-2 RNA Oligonucleotides

**Figure 2:**
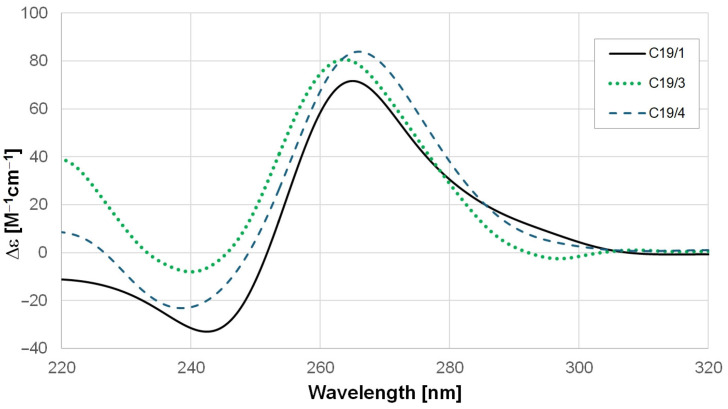
Circular dichroism spectra of C19/1, C19/3, and C19/4 oligomers.

All of the studied RNA oligonucleotides formed GQ structures. Figure 2 shows the circular dichroism (CD) spectra of the C19/1, C19/3, and C19/4 oligomers at pH 7.4 in K-phosphate buffer at 20 °C. As we can see, all of the curves look similar; they have a maximum peak at around 265 nm and a negative peak around 240 nm. Although the spectra are slightly different, all of them are characteristic of the parallel GQ form as it was analyzed with the CD-NuSS server (https://project.iith.ac.in/cdnuss/index.html accessed on 12 January 2024) [51].

**Figure 3:**
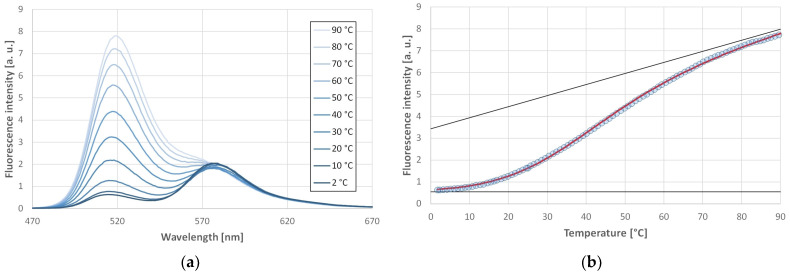
(**a**) Fluorescence spectra of C19/1F at different temperatures. Spectra were measured in pH 7.4 100 mM Na-phosphate buffer; the Na^+^ ion concentration was 170 mM. (**b**) Fluorescence intensity at the donor peak maximum as a function of the temperature. The fitted curve (Equation (7)) is shown in red. The thin black lines show the asymptotes of the fitted function.

This could be confirmed by the FRET experiments. The donor and acceptor were attached to the two ends of the oligonucleotide. The GQ structure was induced in the presence of both K^+^ and Na^+^ ions in case of each investigated oligonucleotide. Figure 3a shows selected fluorescence spectra of C19/1F at selected temperatures in the presence of a 170 mM Na^+^ ion. The increase in the donor fluorescence emission with increasing temperature is indicative of a decrease in the energy transfer, which corresponds to the unfolding of the GQ structure. One can observe almost no fluorescence intensity change in the wavelength characteristic for the acceptor, in contrast to what would be expected from the decreased energy transfer. This occurs for two reasons: First, there might be a small amount of FRET even in the unfolded state. Second, there is a considerable cross-talk between the fluorophores, i.e., the donor emits also at 580 nm, which is the maximum position of the acceptor (see Figure 2d of ref [32], where the emission spectrum of the same donor is presented). The donor emission intensity at 580 nm is around 15% of the intensity at 520 nm. This means that at high temperatures, one measures at 580 nm mainly the donor emission and probably a small acceptor emission from the remaining energy transfer.

The emission intensity of the donor is plotted against the temperature in Figure 3b. The fluorescence signal of the donor can be fitted well with a sigmoid curve (Equation (7)), which is expected from the thermodynamic model described in the Materials and Methods section. This fit allows the determination of the middle point of the unfolding transition (*T*_m_). Similar plots have been obtained in case of oligonucleotides C19/3F and C19/4F as well. Using K^+^ did not change the shape of the curves, although the thermal stability was slightly affected. Similarly, little effect was observed in case of lowering the potassium concentration to the physiological value (140 mM). The obtained parameters are summarized in Table 2 for the studied oligonucleotides stabilized by K^+^ or Na^+^.

### 2.2. Effect of pH on the Stability of SARS-CoV-2 GQs

DNA GQs are known to be moderately pH-sensitive, but no data are available for RNA GQs. Therefore, we investigated the stability of our GQs in the pH range of 6–10. Some of the buffers used in this study are temperature-dependent [52]; therefore, we corrected the pH values. Table 3 shows the corrected values calculated for the transition temperature. One can see a clear stabilization at lower pH values.

By fitting a linear trendline to the pH dependencies, one obtains −5.62 ± 1.00, −1.67 ± 0.49, and −1.89 ± 0,43 °C/pH unit for C19/1F, C19/3F, and C19/4F, respectively.

### 2.3. Volumetric Characterization of SARS-CoV-2 GQs

**Figure 4:**
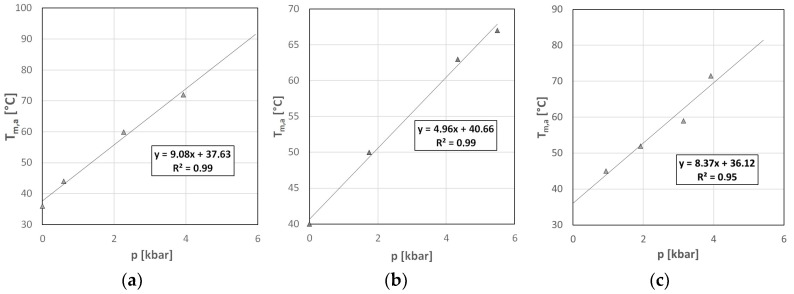
Pressure dependence of the apparent unfolding transition temperatures in case of the (**a**) C19/1F; (**b**) C19/3F; and (**c**) C19/4F oligonucleotides in K-phosphate buffer at pH 7.4. The K^+^ ion concentration is 140 mM. The slope of the fitted line was used to calculate the volume change associated with the folding of the GQ structure.

Any change in the conformation of the molecules, especially of the macromolecules, affects the volume associated with each given molecule. Although these changes are typically quite small (in the range of few percent), we should not forget that the cellular environment is very crowded, containing a high concentration of macromolecules [53,54,55]. In this confined environment, the effect of even slight volumetric changes might be amplified. Since volume as a thermodynamic parameter is conjugated to pressure, one has to apply high pressure to unravel these volumetric aspects. Although pressure is as important a thermodynamic parameter as the temperature, its use is considerably hindered by technical issues. Using our homemade high-pressure diamond cell, we measured the stability curves of all three oligonucleotides in the range of 0–5 kbar (0–500 MPa). Figure 4 shows the pressure dependence of the apparent mid-point of the unfolding transition determined from the D/A plots (see the Materials and Methods section for the details of this evaluation). As can be seen, all of the GQs were stabilized by the pressure. According to the Le Chatelier principle, this stabilization indicates an increased volume of the unfolded oligonucleotide compared to its folded GQ structure. The slopes of the curves in Figure 4 allowed us to determine the unfolding volume changes.

Using the Clapeyron equation, one obtains the following for the volume change at the unfolding:

(1) $$∆V_{u}={\left(\frac{dT_{m}}{dp}\right)}_{pH}\frac{∆H}{T_{m}},$$

where d*T*_m_/d*p* is the slope of the fitted lines in Figure 4, ∆*H* is the enthalpy change of the transition, and *T*_m_ is the unfolding temperature. Here, we emphasize that d*T*_m_/d*p* has to be taken at constant pH. This is necessary since we have to correct our experimental data according to the pressure-induced pH shift (for details, see the Discussion). ∆*H* was obtained from the fit of Equation (7) to the experimental points, as can be seen in Figure 3b.

Table 4 contains the slopes of the *T*_m_-*p* plots. Positive values indicate that pressure is stabilizing the folded GQ form.

### 2.4. Stabilization of SARS-CoV-2 GQs by Ligands

Since human GQs are treated as targets of anticancer drugs, a number of ligands have been developed to stabilize them. These were expected to inhibit the telomerase activity by stabilizing the GQ forms in the telomere region. Although they were developed for DNA GQs, they might also have stabilizing effects in case of RNA GQs. We selected three of these ligands to investigate their effect on the stability of the folded GQ form. The selected ligands are TMPyP4, BRACO19, and PhenDC3. These ligands were added in four-fold excess to the oligonucleotides. Table 5 shows the increase in *T*_m_ upon binding.

**Figure 5:**
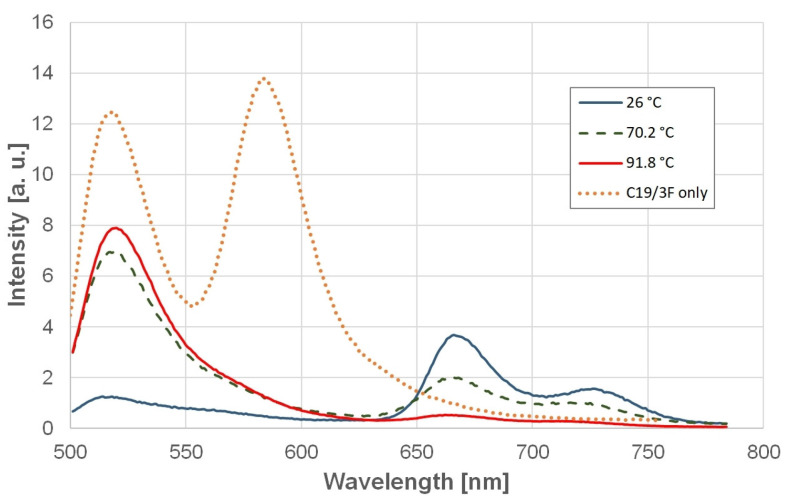
Fluorescence spectrum of C19/3F with TMPyP4 at selected temperatures. One can notice the lack of the acceptor peak (580 nm) and the intensive peaks of TMPyP4 at 670 nm and 730 nm. The latter disappeared at high temperatures. As a control, the spectrum of C19/3F without TMPyP4 at 26 °C (C19/3F only) is also included. The intensity of the red (92 °C) curve is reduced by a factor of three in order to obtain comparable curves.

TMPyP4 stabilized all three oligonucleotides in their GQ form. This stabilization was extremely high; the increase in the donor signal was observed only at the high-temperature end of the experiment, above around 80–90 °C, depending on the oligonucleotide type. The absence of a plateau above the transition prevented the determination of the exact transition temperature with this ligand, although the extremely strong stabilization was obvious. In the case of this ligand, there is an additional energy transfer, namely from the donor to the acceptor and also from the acceptor to TMPyP4. This can be seen from the spectrum of the TMPyP4-bound C19/3F (Figure 5). This is the reason for the lack of acceptor emission. The rapid increase in the donor intensity at high temperatures thus indicates synchronous unbinding of TMPyP4 and unfolding of the GQ structure.

BRACO19 did not show a strong stabilizing effect; in some cases, it seems that there was even a destabilization. PhenDC3 stabilized C19/1F strongly, and C19/4F slightly, but in the case of C19/3F, no stabilization was found.

## 3. Discussion

### 3.1. Stability of GQs Formed by the SARS-CoV-2 RNA Oligonucleotides

The folding of GQ structures can be proven by the CD spectra presented in Figure 2. Earlier studies suggested that RNA quadruplexes can only form parallel topology. The reason for this is that guanine residues in oligoribonucleotides are predominantly in an anti-conformation due to the steric hindrance caused by the 2′hydroxyl group [56,57,58]. In contrast, in DNA, both syn- and anti-conformations can appear, which allows antiparallel and hybrid structures as well [56].

The recorded CD spectra show a positive peak at around 265 nm and a negative peak at 240 nm. These features are characteristic of a parallel GQ structure [59,60]. We also submitted these spectra to the CD-NuSS server (https://project.iith.ac.in/cdnuss/index.html accessed on 12 January 2024), which predicted parallel conformations for C19/1, C19/3, and C19/4 [51].

The stability of the C19/1F oligonucleotide was found to be the smallest from the FRET melting studies. Half of the C19/1 molecules were unfolded already at physiological temperatures. Interestingly, the shortest oligonucleotide, C19/4, shows the highest *T*_m_. It is known for DNA GQs that the length of the loops and the number of G-quartets can influence the stability of the GQs [42,43,61]. All of the GQs formed by these three oligonucleotides consist of two G-quartets, so the number of G-quartets cannot explain the differences in stability.

The type of cation did not influence the stability of the oligonucleotides considerably. According to the common belief published widely in the literature, potassium has a higher stabilizing potential compared to sodium. This is reflected in the *T*_m_ values in case of, e.g., TBA and Htel, where K^+^-stabilized GQs unfold at 10–20 °C higher temperatures than the corresponding Na^+^-stabilized ones [55,62]. In the case of C19 oligonucleotides, there is only a slight difference, and in the case of C19/1F and C19/3F, the Na^+^-stabilized GQ is even a little more stable.

### 3.2. Effect of pH on the Stability of the GQs

DNA GQ structures are known to be fairly pH-insensitive [32]. Interestingly, the i-motif formed by the complementary strand of the GQ sequence is very pH-dependent and needs pH values lower than the physiological one [63,64,65,66]. Our RNA GQs were also found to be quite pH-insensitive, although a small destabilization trend was observed at higher pH values. Their dT_m_/dpH values are smaller than the ones obtained for the DNA GQs of the hepatitis B virus.

### 3.3. Volumetric Characterization of the GQs

As mentioned, pressure is a suitable parameter for studying volumetric aspects of structure formation in larger molecules, since it shifts the equilibrium of the folded–unfolded states, provided they have different volumes.

The determination of the molecular volume is not straightforward; besides the volume of the atoms (*V*_atom_), one has to take into account the volume of the voids (*V*_void_), which are buried in a folded structure, the thermal volume (*V*_thermal_), which is needed for the thermal motions [67], and the volume change caused by the ordering of the solvent around the molecule (Δ*V*_hydration_) [61,67,68]:

*V* = *V*_atom_ + *V*_void_ + *V*_thermal_ + ∆*V*_hydration_ (2)

Folded GQ structures might have a reduced or increased volume compared to their single-stranded form. The volume change (∆*V*_u_) upon unfolding of the GQ is the result of a delicate balance between positive and negative factors. Therefore, the unfolding volume change can be either positive or negative. It is defined as follows:

∆*V*_u_ = *V*_unfolded_ − *V*_folded_ (3)

∆*V*_u_ is positive if the folded state is more compact, i.e., the unfolding increases the volume. In this case, pressure favors the folded form. Since voids appear only in the folded case, this term increases the volume of the folded molecule, i.e., it gives a negative contribution to ∆*V*_u_. On the other hand, the cations lose their hydration shell during the insertion into the folded state, which gives a positive contribution to ∆*V*_u_. Also, the unfolding can change the hydration shell around the molecule, since the solvent-accessible surface is increased in the single-stranded unfolded form compared to the GQ state.

Experimentally, ∆*V*_u_ can be obtained from the pressure-induced shift of the phase transition temperature (*dT*/*dp*) using Equation (1). Although this seems to be simple, there are some technical difficulties to overcome, the most important being the stability of the buffer. Phosphate buffer is quite temperature-stable, which is why it is preferred for temperature stability experiments. However, it shifts considerably with pressure. A pressure increase of 3 kbar causes a pH shift of one pH unit [61,69]. Several pressure studies prefer TRIS buffer, which is fairly pressure-stable. It is a good solution for experiments where only the pressure is varied. However, in our case, we ran temperature scans at different pressures, which would be influenced by the temperature-dependent pH of the TRIS buffer. We decided to correct the *T*_m_ and the experimentally determined *dT*_m_/*dp* values using the known *dpH*/*dp* values of the phosphate buffer. The experimentally measurable pressure dependence of the transition temperature is as follows:

(4) $${\left(\frac{dT_{m}}{dp}\right)}_{\exp}={\left(\frac{dT_{m}}{dp}\right)}_{\mathrm{pH}}+\frac{\partial T_{m}}{\partial pH}\frac{\partial pH}{\partial p},$$

The first term on the right side is the one we must insert into the Clapeyron equation (Equation (1)) in order to obtain the corrected unfolding volume values (∆*V*_u_). The obtained corrected slopes and the corresponding calculated volume changes are summarized in Table 6.

As we can see from Table 6, the unfolding volumes are positive for all of the three oligonucleotides studied in our experiments. This means that the folded form is extremely compact; it lacks large voids. As a consequence of the positive ∆*V*_u_, pressure shifts the equilibrium in the direction of the folded state. One can see that the volume changes are similar for C19/1F and C19/3F, while C19/4F has a roughly twice-higher ∆*V* value. If we take a look at the sequences, C19/4F is the shortest oligonucleotide, which can presumably form the most compact structure. Also noticeable is that the highest *dT*/*dp* was measured for C19/1F, which is the least thermostable, and starts to unfold already at physiological temperatures. All of the GQs consist of two G-quartets, according to the sequence, which contains GG but no GGG repeats. This means that they do not show differences in the volume change caused by the hydration of the cations. Therefore, the different loop sizes can be responsible for the different ∆*V*_u_ values.

Experimentally determined ∆*V*_u_ values for various DNA GQs were summarized in a recent review [61]. These values are in the range of few-times-ten cm^3^/mol, i.e., the volume of a few water molecules. Interestingly, most of the volume change values are negative, but in some cases, especially in the case of viral GQs, one obtained also positive values. As mentioned before, there are positive and negative contributions to the volume change, which partially cancel each other out, and a delicate balance of these contributions governs the sign of the experimental volume change. In our case, the folded state is more compact, and the change in the hydration of the molecule and of the ions contributes to the volume change considerably.

### 3.4. Stabilization of SARS-CoV-2 GQs by Ligands

The stabilization of viral GQs with ligands is a promising method in fighting against viruses. A comprehensive review of viral GQs can be found in refs [30,31]. Although most of the studies in this field were performed in solutions (in vitro), there are also results showing changes in the viral metabolism. Ruggiero et al. showed that TMPyP4 interacts with GQs of the herpes simplex virus, and inhibits polymerase progression in vitro [70].

Martino [71] studied the interaction of TMPyP4 with Htel and detected a conversion of the hybrid conformation to an antiparallel structure due to interaction with TMPyP4 in dilute solutions, but not in crowded ones.

Our finding that TMPyP4 stabilizes GQs of the SARS-CoV-2 genome effectively is in agreement with the results found by Qin et al., who found TMPyP4 treatment to be effective against COVID-19 in an animal model [38]. A similar compound, which binds to GQs with smaller activity, had no effect against the disease, which proves the role of GQs in the antiviral effect.

There is not much direct information about the interaction of RNA GQs with ligands. Oliva et al. compared the binding of berberine to C19/4 and to human telomere [46]. The binding constant was less for C19/4, especially in presence of potassium ion.

In some cases, destabilization is also obtained due to ligand binding. Oblak et al. investigated the phase space of the Htel, wherein they also obtained a range where the ligand binding stabilized the intermediate state against the folded GQ one [72]. A similar effect could be behind the C19/3F results found in the present study, where slight destabilization was observed due to the binding of BRACO19 and PhenDC3.

Our results also showed a relatively small stabilization compared to our earlier study, wherein we proved considerable stabilization of GQs of the hepatitis B virus using the same three ligands [9].

This means that effective stabilization of RNA GQs requires special new ligands, besides TMPyP4, which was found to be an effective stabilizer.

## 4. Materials and Methods

### 4.1. Materials

Table 1 shows the sequences of the oligonucleotides studied. They are the four oligonucleotides that were found to be GQ-prone by Panera et al. [47]. For the Förster resonance energy transfer (FRET) studies, we used labeled oligonucleotides. Since guanine tends to quench the fluorescence of the used fluorophores (FAM and TAMRA), one base was added to both ends of the oligonucleotides from the original sequence of the virus to increase the guanine–fluorophore distance. The labeled oligonucleotides were ordered from IDT DNA (Coralville, IA, USA) and Eurofins (Ebersberg Germany).

Other materials like the buffer compounds and salts were obtained from Sigma (now Merck). Milli-Q water was used to prepare all the solutions.

Buffers were pretreated by adding RNAsecure (Invitrogen, Waltham, MA USA) in a 1/25 volume ratio. This solution was heat-treated at 60 °C for 10 min right before the measurement. The oligonucleotide stock solution was added after the cooling of the buffer solution.

RNA oligonucleotide solutions were prepared in K-phosphate or Na-phosphate buffers, which also provided the ion for the stabilization of the GQs. Unless otherwise stated, the buffers had a pH value of 7.4 and a concentration of 100 mM.

The oligonucleotide concentration was 1 µM in all fluorescence experiments, except for the pressure studies, where the small sample size was compensated for by increasing the oligonucleotide concentration to 10 µM. For the pH dependence assessments, we used additional buffers: phosphate, TRIS, and CAPS buffers, with pH values of 6.0, 8.8, and 10.1, respectively, at 20 °C. The pH was corrected during the evaluation using the temperature dependence of pH reported in the literature [52,73,74]. In these cases, 140 mM KCl was added to ensure the same cation concentration as it was in the case of the phosphate buffers. Pressure dependence of pH was taken into account according to Kitamura et al. [69]. All of the buffers contained 0.1 mM EDTA.

### 4.2. Methods

***Fluorescence experiments*** were performed as described earlier in detail [9,75]. Fluorescence spectra were recorded with a Fluorolog-FL3 fluorimeter (Horiba Jobin Yvon, Longjumeau, France). It was equipped with a programmable temperature-controlled cell (DI instruments, Budapest, Hungary). A temperature ramp of 0.2 °C/min was set for the temperature scans. Since the spectra were recorded every 5 min, we had spectra in 1 °C intervals. The temperature in the cuvette or in the pressure cell was measured directly using a thermocouple connected to an HH802U-type thermometer (Omega, Irving, TX, USA). Its software allowed us to record the temperature every 30 s. The exact temperature of the measurement was obtained using this log file and the time stamp of the spectrum.

The fluorescence spectra were recorded with λ_exc_ = 455 nm. The light source of the Fluorolog-FL3 fluorimeter was replaced by a high-intensity LED, which was driven by a controlled power supply [55]. This gave a very stable intensity with <0.01% relative standard deviation.

The spectra were evaluated by determining the position and intensity of the fluorescence peaks using the Savitzky–Golay peak-finding algorithm [76,77].

***Förster Resonance Energy Transfer*** (FRET) is sensitive to the distance of the two fluorophores. We used FAM and TAMRA as the donor and acceptor. The folded GQ structures show a marked FRET effect: exciting the donor, we see intense emission from the acceptor, while in the unfolded random-coil RNA strands, only the donor emission can be seen, because the acceptor is too far for FRET. The Förster distance, where the energy transfer reduces to 50%, is 5.5 nm in the case of the fluorophore pair we used. The extended chain of the shortest oligonucleotide (C19/4) is 5.2 nm, but the linker of the fluorophores adds ca. 1nm at each side, which will well exceed the Förster distance. This means that the lack of FRET actually indicates the single-stranded conformation of the oligonucleotide.

***Thermodynamic model of unfolding***: The unfolding of the GQ structure was described using a two-state thermodynamic model, which gives the ratio of the thermodynamical weights *w*_GQ_ and *w*_SS_ of the folded GQ and of the single-stranded unfolded states with the following relation:

(5) $$\frac{w_{SS}}{w_{GQ}}=e^{-\frac{∆G}{R\cdot T}},$$

where ∆*G* is the Gibbs free energy difference between the folded and unfolded states: ∆*G* = *G*_unfolded_ − *G*_folded_. *R* is the gas constant and *T* is the temperature. *w*_GQ_ is the relative amount of molecules folded into the GQ state, i.e., the number of GQ oligomers divided by the total number of the oligomers. A similar definition applies for *w*_SS_.

Since *w*_GQ_ + *w*_SS_ = 1, the amount of unfolded oligonucleotides can be written as follows:

(6) $${\;}_{w_{SS}\left(T\right)=\frac{1}{1+e^{\frac{∆H}{R}\cdot (\frac{1}{T}-\frac{1}{T_{m}})}}}\;\;$$

Since the donor fluorescence intensity is considerably higher in the single-strand state than in the folded one, it can be fitted with the following equation:

(7) $$y\left(T\right)=a+b\cdot T+\frac{∆a+∆b\cdot T}{1+e^{\frac{∆H}{R}\cdot (\frac{1}{T}-\frac{1}{T_{m}})}},$$

Here, we assume a linear temperature dependence of the intensity in both states. The parameters *a*, *b*, Δ*a*, Δ*b* describe these dependencies. Δ*H* is the enthalpy change during the unfolding transition. *T*_m_ is the midpoint of the unfolding transition. Fitting the data with Equation (7) was used to determine the *T*_m_ and Δ*H* parameters. Nonlinear fitting was conducted using the Origin^®^ program (OriginLab Corporation Northampton, MA, USA), which was able to provide the standard error of the parameters as well. We used these error values to calculate the confidence intervals using the Student’s *t*-value for a 95% confidence level. We would like to emphasize that this is the correct statistical evaluation. Special care has to be taken if one compares different publications, since several publications simply give the standard error, which shows the experiment as being more precise, even if it is not.

Regarding the pressure experiments, where the upper or lower side of the transition curve could not be fitted unequivocally, we determined the apparent transition point (*T*_m,a_), which is the temperature at which the intensity ratio of the donor and acceptor emission (*D/A*) equals 2. This value was chosen since the *D*/*A* curve starts at 0.5 for the folded GQ, while this value is around 3.5 for the unfolded one. The value (*D*/*A*)_m_ = 2 can therefore be treated as the apparent middle point of the transition. In the subsequent calculations, only the pressure dependence of the *T*_m,a_, and not the actual *T*_m_ values are used; therefore, the choice of the value (*D/A*)_m_ does not influence the results. For the C19/4F oligonucleotide, we used (*D/A*)_m_ = 2.5, since the *D/A* values start and end at higher values.

***Ligand-binding stabilization experiments***: Ligands were added in four-fold excess to the actual oligonucleotide solution. The final concentrations were 4 μM for the ligands (TMPyP4, BRACO19, or PhenDC3) and 1 μM for the oligonucleotide. The ligands and RNAsecure (Invitrogen, Waltham, MA, USA) were added to the buffer solution before the heat treatment of the RNAsecure (Invitrogen, Waltham, MA, USA). Only the RNA oligonucleotides were added after the heat treatment. This order provides the best protection against naturally occurring RNase enzymes, since every component is heat-treated before the addition of the RNA sample, and no untreated components need to be added afterwards.

***High-pressure experiments***: For the high-pressure experiments, a homemade reflection-mode diamond anvil cell was adopted into the sample holder. The diamond anvil was glued into a carbide plate, which was purchased from Almax-easyLab (Diksmuide, Belgium). The pressure was measured by recording the ruby fluorescence [78]. A small ruby chip was placed into the pressure cell, and it was excited by a green HeNe laser (Coherent, USA). The emitted light was detected with a CCD camera (Andor, Belfast, UK) attached to a THR1000 monochromator (Jobin Yvon, now Horiba, Longjumeau, France).

***CD measurements***: CD experiments were performed on a Jasco J-815 spectrometer (Jasco LTD, Tokyo, Japan) in Hellma cuvettes with path lengths of 0.1 cm. The slit was set to 2 nm, the registration speed was set to 50 nm/min, and 5 scans were averaged in each experiment. The temperature was set to 20 °C using a Jasco CDF-426L Peltier thermostat. The concentration of the oligos was between 8 and 15 μM as measured by the absorbance at 260 nm. The spectra shown in Figure 2 are all scaled to a 10 μM concentration in order to obtain comparable plots.

## 5. Conclusions

Our experiments showed that three of the studied RNA oligonucleotides of the SARS-CoV-2 genome formed GQ structures. They showed different temperature stability levels, with C19/1F being the least stable. They showed very moderate pH dependence. All of them could be stabilized by pressure, indicating a positive unfolding volume. This allows us to conclude that the oligonucleotide C19/4F has the most compact form. Marked stabilization of all of the GQs was achieved by TMPyP4, while BRACO19 had hardly any stabilization. PhenDC3 stabilized C19/1F and C19/4F only.

Although these stabilizing effects are smaller than those found earlier for hepatitis B, we hope that these results can lead to better understanding of the structure and stabilization of RNA GQs and can contribute to the fight against viral infections, including that of SARS-CoV-2.

## Figures and Tables

**Table 1:** Sequences of the oligonucleotides used in our experiments. The oligonucleotides were numbered after their appearance in ref [47].

Name	Sequence (5′–3′)	Length	Position	Gene
C19/1	GG UAU GUG GAA AGG UUA UGG	20	13385	ORF1ab
C19/1F	Fam-CGG UAU GUG GAA AGG UUA UGG C-Tamra	22	13384	
C19/3	GG UGU UGU UGG AGA AGG UUC CGA AGG	26	1574	ORF1ab
C19/3F	Fam-AGG UGU UGU UGG AGA AGG UUC CGA AGG U-Tamra	28	1573	
C19/4	GG CUG GCA AUG GCG G	15	28903	N
C19/4F	Fam-UGG CUG GCA AUG GCG GU-Tamra	17	28902	

**Table 2:** Parameters of the unfolding transition of the oligonucleotides studied in the presence of K^+^ and Na^+^ ions. *T*_m_ is the mid-point of the unfolding transition, where the GQ structure unfolds. ∆*H* is the enthalpy change associated with this transition. The parameters were determined from fitting experimental intensities with Equation (7). For the fitted parameters, 95% confidence intervals are indicated as follows: fitted parameter ± 2 · error of the fitted parameter.

Name	Cation	Cation Conc. (mM)	*T*_m_ (°C)	∆*H* (kJ/mol)
C19/1F	K^+^	140	37.0 ± 1.0	54.4 ± 0.6
	K^+^	170	36.6 ± 0.4	95.6 ± 2.0
	Na^+^	170	37.5 ± 0.1	63.9 ± 0.8
C19/3F	K^+^	140	47.6 ± 0.4	107 ± 3
	K^+^	170	49.6 ± 0.7	119 ± 6
	Na^+^	170	53.8 ± 0.5	106 ± 4
C19/4F	K^+^	140	61.9 ± 0.3	129 ± 2
	K^+^	170	57.9 ± 0.3	151 ± 5
	Na^+^	170	53.7 ± 0.9	117 ± 5

**Table 3:** pH dependence of the temperature stability of the C19/1F, C19/3F, and C19/4F oligonucleotides.

C19/1F		C19/3F		C19/4F	
pH	*T*_m_ [°C]	pH	*T*_m_ [°C]	pH	*T*_m_ [°C]
5.91	53.7	5.91	51.7	5.83	58.6
7.35	40.2	7.32	49.0	7.24	58.1
8.36	36.6	7.98	50.3	7.82	55.9
10.02	29.8	9.89	44.8	9.83	51.1

**Table 4:** Volumetric parameters calculated from high-pressure experiments of K^+^-stabilized C19 oligonucleotides. Slope values of the experimentally determined *T*_m,a_-*p* curves and the calculated unfolding volumes were calculated using the Clapeyron equation. The 95% confidence intervals are given for the fitted parameters.

Determined Parameter	Oligonucleotide			Unit
	C19/1F	C19/3F	C19/4F	
(d*T*/d*p*)_exp_	9.1 ± 2.9	5.0 ± 1.1	8.4 ± 5.8	°C/kbar

**Table 5:** Stabilizing effect of TMPyP4, BRACO19, and PhenDC3 on C19 GQs. The GQ solutions contained a 140 mM K^+^ ion.

Ligand	Oligonucleotide					
	C19/1F		C19/3F		C19/4F	
	*T*_m_ [°C]	∆*T*_m_ [°C]	*T*_m_ [°C]	∆*T*_m_ [°C]	*T*_m_ [°C]	∆*T*_m_ [°C]
none	37.0	-	47.6		61.9	
TMPyP4	>80	>40	>90	>40	>90	>40
BRACO19	41.7	5.3	48.5	1	55.4	−6.5
PhenDC3	68.5	31.5	46.6	−1	68.7	6.8

**Table 6:** Volumetric parameters calculated from high-pressure experiments of K+-stabilized C19 oligonucleotides. Slope values of the *T*_m,a_-*p* curves and the calculated unfolding volumes were calculated using the Clapeyron equation. For the ∆*V*_u_ values, the 95% confidence interval is indicated.

Parameter	Oligonucleotide			Unit
	C19/1F	C19/3F	C19/4F	
measured d*T*/d*p*	9.08	4.96	8.37	°C/kbar
pH-corrected ∂*T*/∂*p*	10.8	5.46	8.94	°C/kbar
∆*V*_u_	19 ± 7	21 ± 6	36 ± 26	cm^3^/mol

## Data Availability

Original data can be obtained from the corresponding author on request.
